# Predicting Contribution in High Achieving Black and Latinx Youth: The Role of Critical Reflection, Hope, and Mentoring

**DOI:** 10.3389/fpsyg.2021.681574

**Published:** 2021-07-07

**Authors:** Edmond P. Bowers, Candice W. Bolding, Luke J. Rapa, Alexandra M. Sandoval

**Affiliations:** ^1^College of Behavioral, Social and Health Sciences, Clemson University, Clemson, SC, United States; ^2^College of Education, Clemson University, Clemson, SC, United States

**Keywords:** contribution, positive youth development, critical consciousness, hope, mentoring, youth of color

## Abstract

Contemporary approaches to adolescent development are framed by positive youth development models. A key outcome of these models is that healthy and positively developing youth are more likely to contribute to their family, schools, and communities. However, little work on contribution and its antecedents has been conducted with youth of color. As high achieving youth of color often become leaders in their communities, it is important to consider malleable predictors of contribution within this population. Therefore, through a cross-sectional design, we examined the relations between youth critical reflection, hopeful future expectations, and mentoring relationship quality and youth contribution in a sample of 177 youth of color (60% Black, 40% Latinx) attending an afterschool college preparation program at six sites around the U.S. Results indicated that youth critical reflection, hopeful future expectations, and mentoring relationship quality significantly predicted contribution. Exploratory analyses suggested that these relations were significant for Black youth but not Latinx youth. Implications of these findings for future scholarship are discussed.

## Introduction

Strengths-based, positive youth development (PYD) approaches framed by relational developmental systems (RDS) models of human development have come to the fore of the contemporary study of adolescent development (Lerner et al., 2015). As derived from RDS models, PYD approaches to adolescent development are based on several fundamental precepts. These precepts include the following: (1) all young people have strengths that may be capitalized on to promote thriving; (2) resources for healthy and positive development can be found in the contexts in which youth are embedded; and (3) when the strengths of youth are aligned with the resources found in contexts of youths' lives, youth are more likely to thrive (Lerner et al., 2013, 2015). In turn, a key outcome of these PYD processes is that thriving youth are more likely to be social change agents and contribute to their families, schools, and communities (Lerner, 2004; Hershberg et al., 2015). Contributing youth tend to possess an outward-oriented ideology and act in ways that strengthen the contexts in which they live (Lerner, 2004; Geldhof et al., 2015). Prior research has indicated that youth contribute to these settings in diverse ways, for example, at home helping parents by babysitting younger siblings, at school by participating in student government, and in the community by volunteering at soup kitchens; youth can also contribute through more civically-engaged actions, such as supporting social causes through letter writing, protesting, and activism (Zaff et al., 2010; Hershberg et al., 2015). Youth contribution is critical to the betterment of society and is predictive of adult well-being and success (Chan et al., 2014; Metzger et al., 2018). Therefore, identifying individual strengths and contextual resources that influence contribution in adolescence is a key goal of practitioners, policy makers, and scholars.

There are several models based on RDS that have been used to study PYD (Lerner et al., 2015). The Lerner and Lerner Five Cs model of PYD is one of the most empirically supported frameworks for PYD (Heck and Subramaniam, 2009; Lerner et al., 2015). As the Five Cs model is explicitly founded on RDS principles, the research testing this model frequently explores how youth strengths (e.g., intentional self-regulation, hopeful future expectations, critical reflection) can be aligned with resources from key contexts of youth lives (e.g., schools, youth programs) to promote thriving (as marked by the Five Cs of competence, confidence, connection, character, and caring) and contribution in youth (Lerner et al., 2013, 2015). Much of the existing empirical evidence on the Five Cs model is based on data drawn from the 4-H Study of PYD (Lerner et al., 2005; Bowers et al., 2014). The 4-H Study of PYD is a longitudinal study of ~7,000 U.S. adolescents explicitly designed to test the Five Cs model of PYD. These studies have identified several individual strengths and contextual resources that predict youth contribution, including hopeful future expectations (Schmid and Lopez, 2011; Schmid et al., 2011), intentional self-regulation (Zimmerman et al., 2008; Bowers et al., 2011; Schmid et al., 2011), parenting (Lewin-Bizan et al., 2010), and participation in youth development programming (Mueller et al., 2011; Agans et al., 2013). This evidence, however, has been derived from relatively homogenous samples of White youth (Bowers et al., 2014; Spencer and Spencer, 2014; Travis and Leech, 2014; Hershberg et al., 2015).

The findings based on this work may not be generalizable to youth of color as the historical, social, and structural inequities that often create marginalizing systems for these youth may affect *which* individual and contextual factors—as well as which relations among those factors—are linked to thriving and contribution among these youth (Garcia-Coll et al., 1996; Spencer and Spencer, 2014). Such differences could be systemic because youth of color are often excluded from or do not have access to contexts where acts of contribution occur, such as schools, shelters, and youth programs (Ginwright and Cammarota, 2002; Hope and Jagers, 2014). To promote thriving in all youth, it is therefore critical to identify culturally and contextually relevant strengths and resources that foster their positive development (Travis and Leech, 2014; Clonan-Roy et al., 2016).

In addition, little consideration has been given for how these PYD processes may differ among diverse youth of color. Multiple overlapping systems of marginalization may lead to differentiated experiences for individuals across dimensions of marginalization (Lerner et al., (In press); Crenshaw, 1990; Godfrey and Burson, 2018). Whereas, prior research has examined types of contribution within samples of “at-risk” youth of color (Chan et al., 2014) and among urban youth of color (Christens et al., 2018), the effects of key facets of marginalization within these groups have not been considered. For example, when exploring the heterogeneity of experiences among youth of color, one critical way the lives of these youth may differ greatly is related to their ability to succeed within school settings (Rowley and Moore, 2002; Nasir et al., 2009; Wright, 2009; Chambers, 2011).

Indeed, youth of color who are academically high achieving may navigate the marginalizing system of school (among other marginalizing systems) in qualitatively different ways than their peers who are less academically successful (Flores-González, 2002; Carter, 2008; Chambers, 2011). Therefore, the individual and contextual resources key to youth contribution may be qualitatively different for high achieving youth of color compared to their counterparts (Nasir et al., 2009; Hershberg et al., 2015). Although academic competence and success is one key marker of PYD (e.g., Bowers et al., 2010; Geldhof et al., 2014), a more comprehensive approach to understanding the well-being of academically high achieving youth of color is needed to understand, if not enhance, the likelihood of their success beyond the school setting (e.g., Olszewski-Kubilius and Clarenbach, 2012; Plucker et al., 2015).

Theoretical and empirical evidence points to several factors that may be influential in contribution among high achieving youth of color along several cognitive, emotional, and behavioral dimensions important to contribution among youth experiencing marginalization. These strengths include critical reflection of how power and privilege operate within inequitable societies; self-efficacy and hope to promote change in one's community; and the guidance of supportive and caring adults (Ginwright and James, 2002; Christens, 2012; Diemer and Rapa, 2016; Diemer et al., 2016; Christens et al., 2018; Heberle et al., 2020; Rapa et al., 2020a). The individual strengths of critical reflection and hope and the contextual resource of mentoring have also been found to promote the well-being and positive development in high achieving youth of color in diverse ways (Sellers et al., 1998; Hébert and Reis, 1999; Cook, 2000; Muller and Ellison, 2001; Flores-González, 2002; Smith, 2003; Reis et al., 2004; Antrop-González et al., 2007; Carter, 2008; Olszewski-Kubilius and Clarenbach, 2012; Williams and Bryan, 2013; Bowers et al., 2020). For example, in a prior study of high achieving youth of color living in urban communities, Bowers et al. (2020) found that critical reflection and mentoring relationship quality were linked to PYD as measured by the Five Cs. However, few studies have examined the extent to which these factors—critical reflection, hope, and mentoring—play a role in contribution among high achieving youth of color.

High achieving youth of color residing in urban contexts have been shown to often take on leadership roles within their communities (Howard et al., 2016), and their successes serve as “counterstories” to the more deficit-oriented perceptions and approaches that have predominated narratives and research with youth of color (Cabrera, 2013; Dill, 2017). Therefore, identifying the constellation of factors linked to contribution among high achieving youth of color can not only further illuminate their strengths, but also provide guidance for future scholarship on the description, explanation, and optimization of their development. Only a few studies have examined high achieving youth of color residing in urban contexts and the strengths and resources that influence their thriving (Hébert and Reis, 1999; Flores-González, 2002; Antrop-González et al., 2007; Carter, 2008; Henfield, 2013; Williams and Bryan, 2013; Bowers et al., 2020). In the present study, we address this gap by taking a PYD approach to determine the extent to which the individual strengths of critical reflection and hopeful future expectations and the contextual resource of mentoring relationship quality predict contribution in a sample of academically high-achieving urban youth attending an afterschool college preparation program in six U.S. cities.

### Critical Reflection as an Individual Resource Supporting Contribution

Critical reflection is the analysis of societal inequities as unjust and the linking of those societal inequities to sociohistorical systems of oppression and marginalization (Freire, 1973; Diemer et al., 2016). A growing body of work has examined the positive effects of critical reflection on well-being and positive outcomes for youth experiencing marginalization (for review, see Heberle et al., 2020). Within this body of work, scholars have sought to identify how critical reflection leads to critical action, civic or political engagement, and community engagement among youth of color (Westheimer and Kahne, 2004; Diemer and Rapa, 2016; Heberle et al., 2020)—all of which may be reflected in measures of contribution. In brief, the more youth experiencing marginalization critically reflect on and analyze societal inequities, the more likely they may be to engage in activities that bring about systemic and social change and contribute to fostering more just outcomes for their families, their schools, and their communities.

For example, drawing on data derived from a nationally-representative sample, Diemer and Rapa (2016) found that critical reflection increased expected voting behavior among Latinx youth. Similarly, higher levels of critical reflection among Black youth were linked to higher levels of participation in conventional political action, for example volunteering for political campaigns. Additionally, critical reflection and analysis of sociopolitical systems and government institutions have been found to increase marginalized youths' engagement in civic activities, including volunteering and community service (Hope and Jagers, 2014). The exploration of the extent to which critical reflection is associated with high achieving youth of color's contribution will add nuance to our understanding of how critical reflection relates to this aspect of PYD and will provide new insights for programs seeking both to develop youth of color's ability to analyze societal inequities and to promote civic action.

### Hope as an Individual Resource Supporting Contribution

PYD research defines hope as comprising three components: (1) intentional self-regulation; (2) positive future expectations; and (3) connectedness (Callina et al., 2015). Working together, these three components support positive development (Schmid et al., 2011; Callina et al., 2015). Studies have found that hope is associated with numerous positive outcomes such as adaptive coping mechanisms, more positive thoughts, more positive perceptions of stressful events, and higher motivation to achieve goals (Affleck and Tennen, 1996; Snyder et al., 2000; Kenny et al., 2010; Roesch et al., 2010). Additionally, components of hope have been associated with youth's contribution and civic engagement (Callina et al., 2015). Whereas the majority of research on hope has focused on White, middle class youth, there is evidence that similarities exist between White youth and youth of color for the benefits of having hope (Chang and Banks, 2007; Roesch et al., 2010). That is, research examining the impact that hope has on positive outcomes for youth of color indicates that higher levels of hope are associated with positive outcomes for populations experiencing marginalization.

For example, Roesch et al. (2010) found that youth of color who possessed a high level of hope used more coping strategies (i.e., problem solving, positive thinking, religious coping, etc.) when dealing with daily stressors. Additionally, Kenny et al. (2010) found that higher levels of hope among urban youth were predictors of achievement-based beliefs. Although there is a small body of work that examines hope and positive outcomes for youth of color, there is less that examines how hope promotes contribution in youth of color. In one of the few studies that explores these relations, Christens et al. (2018) examined the association between critical hopefulness and components of sociopolitical development (e.g., civic engagement). In their framework, critically hopeful individuals possess the ability to remain hopeful even as their critical awareness of systemic inequities increases (Christens et al., 2018). Study results suggested that urban high-school-aged youth with higher levels of critical hopefulness were more likely to participate in civic engagement. Additional research on the association between hope and contribution among youth of color can add to the growing body of evidence identifying culturally relevant strengths among youth of color and can provide potential insight into factors that youth programs may find malleable as they work to support the positive development of diverse youth.

### Mentoring as a Contextual Resource Supporting Contribution

Mentoring relationships have been identified as one of the most impactful resources for promoting healthy and positive development in young people (Rhodes and Lowe, 2009; Bowers et al., 2012, 2015; Li and Julian, 2012), including youth of color generally (Erickson et al., 2009; Raposa et al., 2018) and high-achieving youth of color specifically (Hébert and Reis, 1999; Cook, 2000; Flores-González, 2002; Williams and Bryan, 2013; Bowers et al., 2020). Notably, youth often develop mentoring relationships through afterschool youth development programs (Lerner et al., 2014). Consistent with findings from the 4-H Study, engagement in youth development programming has been linked to contribution on youth of color; however, programs effective at promoting contribution among youth of color are often framed by social justice youth development (SJYD) principles (Ginwright and Cammarota, 2002; Ginwright and James, 2002). SJYD programs emphasize youth empowerment through exploring and confronting marginalization. In programs grounded in SJYD approaches, adult facilitators guide youth as they develop critical reflection skills, self-efficacy to enact change in their communities, and action plans to address local inequities and injustices. Thus, youth participation in programs framed by SJYD principles are likely to engage in contextually relevant and meaning contributions (Hershberg et al., 2015). Similarly, contemporary approaches to mentoring posit mentoring relationships can be leveraged to promote youth contribution (Albright et al., 2017; Sánchez et al., 2021). For example, through critical mentoring relationships (Weiston-Serdan, 2017), mentors partner with youth to leverage youth strengths in empowering young people to contribute by addressing systemic issues and promoting their own well-being. Through relationships formed in SJYD and critical mentoring programs, youth build a sense of efficacy and a sense of community to effect social change (Camino and Zeldin, 2002; Liang et al., 2013). In general, however, there is scant research on the role of mentors in promoting contribution. Most of the work based on these contemporary frameworks to youth development has been qualitative studies of social-justice oriented youth programs with missions to promote critical reflection and community engagement. Examining how mentors in other programs—for example, programs focused on outcomes such as academic success, college preparation, or career readiness—support contribution among high achieving youth of color is important to building our understanding of how to promote positive community action within diverse youth settings.

### The Present Study

**Figure 1:**
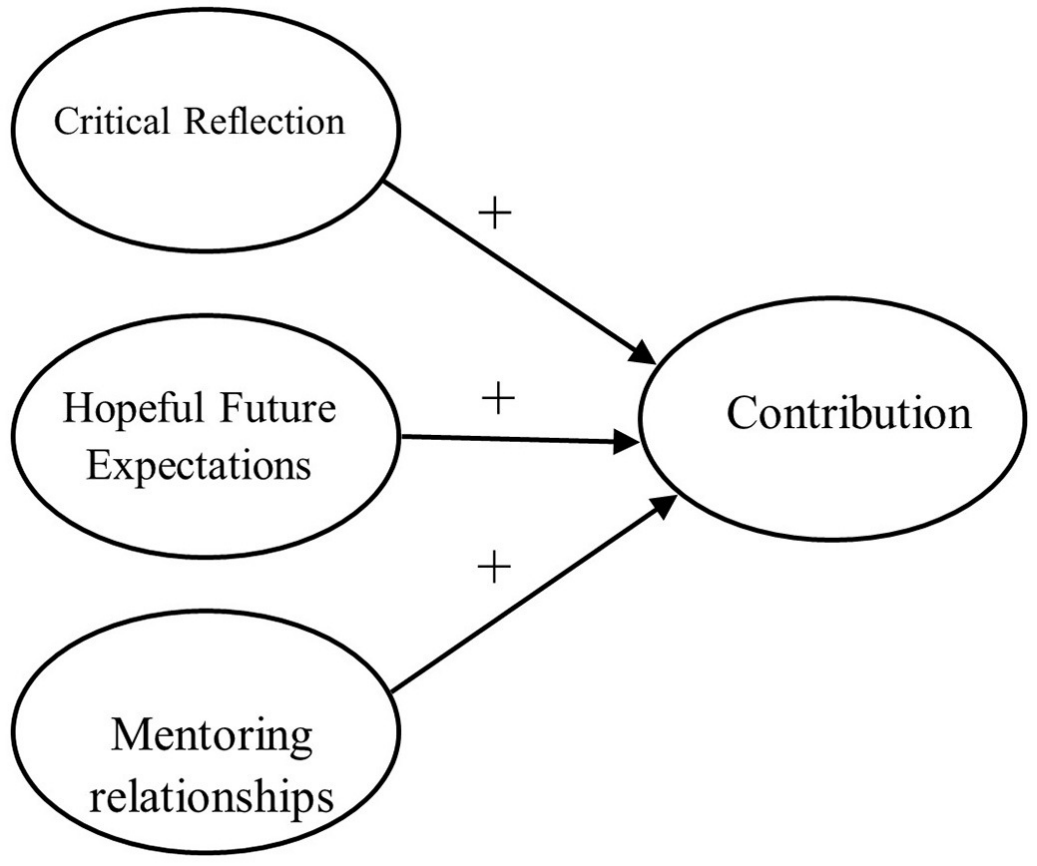
Conceptual model. Positive hypothesized relations are depicted (+) between youth's critical reflection, hopeful future expectations, and mentoring relationships and their contribution.

As noted, most studies of contribution from a PYD perspective have been based on largely White middle-class samples; therefore, the antecedents identified in this existing body of work may not be applicable to more diverse samples of youth. From an RDS perspective, it is important to identify culturally and contextually relevant predictors of contribution to understand more fully the processes of thriving across diverse youth and to identify potential means to optimize the likelihood of thriving among these youth. This work was thus guided by the following central research question: To what extent do critical reflection, hopeful future expectations, and mentoring relate to contribution in academically high-achieving youth of color living in urban contexts? Given extant research in this area, though scant in terms of specific studies examining these relations with high-achieving youth of color, we expected that critical reflection, hopeful future expectations, and mentoring would each positively relate to contribution (see Figure 1). Given the dearth of empirical evidence exploring these relations among this study's population of interest, we made no hypotheses about the strength of these associations or about which predictor might relate most strongly to contribution; we expected them to be positively related nonetheless.

We had a secondary interest in examining the extent to which associations between critical reflection, hopeful future expectations, mentoring, and contribution may differ based on ethnic-racial identification (i.e., Black versus Latinx). However, due to limitations with subgroup sample size for these two ethnic-racial identification groups (see the Participants section below), we considered any analyses related to this secondary interest to be exploratory.

## Materials and Methods

The sample of youth in this study included youth of color residing in urban contexts who were participants in Boys Hope Girls Hope (BHGH) afterschool college preparation program sites (referred to as BHGH Academy Programs) located in six cities across the United States (Phoenix, AZ; Aurora, CO; Detroit, MI; Cleveland, OH; San Francisco, CA; and St. Louis, MO). BHGH also offers residential programming at additional sites around the country and internationally, but the present study focused on BHGH afterschool programming. BHGH “provides holistic poverty intervention for motivated youth with demonstrated need” (Boys Hope Girls Hope, 2020a). Most youth in BHGH come from families experiencing poverty and a lack of academic opportunities within communities affected by violence, substance abuse, and mental health issues, with 72% of youth in the program coming from families whose household income falls below the US poverty line (Boys Hope Girls Hope, 2020b). Although guided by the same mission and overall program model, the various BHGH afterschool sites employ diverse strategies and approaches in delivering the program. Programs at each site provide youth with college readiness activities, mentoring, connections to college and career paths, and support during key life transitions.

Based on prior definitions in the literature, the study sample was a group of academically high-achieving young people; that is, they excelled in school and maintained above-average academic achievement (Hébert and Reis, 1999). Although procedures differed somewhat across sites, youth were required to complete an application process to join the BHGH afterschool program, which included a personal statement, essay questions, and an interview with staff.

Data for this study were derived from a larger project in which 256 program youth (61.3% female/38.7% male) completed questionnaires in Fall 2017 (Bowers et al., 2020). The number of youth who participated from each site reflected the unique structure, format, and model of program delivery at each site (Phoenix, AZ: 41 participants; Aurora, CO: 34 participants; Detroit, MI: 44 participants; Cleveland, OH: 91 participants; San Francisco, CA: 20 participants; and St. Louis, MO: 26 participants). Participants ranged in age from 11 to 18.5 (*Mage* = 15.23, *SD* = 1.94) and were overwhelmingly youth of color, predominantly Black (46.9%) or Latinx (33.6%). The youth were students in middle school and high school at the time of data collection. Overall, the average participant had been enrolled in BHGH Academy programming for 3 years and attended the program for 5 h per week.

Of the 256 youth who participated, 221 reported that they had at least one adult mentor (79.6%). Of these 221 youth who reported having a mentor, 61.5% identified as female. Nearly half (48.4%) reported their ethnic-racial identification as Black, and nearly one-third (31.7%) reported their ethnic-racial identification as Latinx. Given that in the present study we sought to examine the experiences of Black and Latinx youth, specifically, we removed any youth who identified with an ethnic-racial group other than Black or Latinx. This process provided us with a sample size of 177 youth (60 % Black, 40% Latinx) in our final analytic sample.

### Measures

#### Critical Reflection

Critical reflection was assessed using the eight-item Perceived Inequality subscale of the Critical Consciousness Scale (Diemer et al., 2017). Sample items include “Certain racial or ethnic groups have fewer chances to get a good high school education;” “Certain racial or ethnic groups have fewer chances to get good jobs;” and “Poor people have fewer chances to get ahead.” Participants responded on a five-point Likert-type scale ranging from 1 (strongly disagree) to 5 (strongly agree). Cronbach's alpha for the critical reflection scale in the current sample was α = 0.96 and the mean inter-item correlation was 0.738.

#### Hopeful Future Expectations

Hopeful future expectations were assessed using six items from a measure used previously in the 4-H Study of Positive Youth Development (Bowers et al., 2014) and described by Schmid et al. (2011). Items assessed participants' expectations that they will experience certain situations later in life. Participants were provided the following prompt: “Think about how you see your future. What are your chances for the following?” and then provided with a number of items reflecting different future life situations or circumstances. Items involved helping other people, being healthy, being safe, graduating from college, having a job that pays well, and having friends you can count on. Responses were provided on a Likert-type scale ranging from 1 (very low) to 5 (very high). Higher scores reflect higher personal expectations that positive future outcomes will occur in one's life. Cronbach's alpha in the current sample was α = 0.96 and the mean inter-item correlation was 0.473.

#### Mentoring Relationship Quality

Mentoring relationship quality was assessed using eight items from Rhodes et al. (2005) Youth-Mentor Relationship Questionnaire. Participants were asked to think about the adult mentor in the program to whom they were closest at the time of the data collection. Youth were informed that “a mentor is anyone who is more experienced and who helps you. For example, an adult volunteer or staff member.” Sample items include “I can trust this person” and “This person has good ideas about how to solve problems.” Participants responded on a four-point Likert-type scale ranging from 1 (not true at all) to 4 (very true). Higher scores reflected perceptions of better mentoring relationship quality. Cronbach's alpha in the current sample was α = 0.71 and the mean inter-item correlation was 0.276.

#### Contribution

Contribution was assessed using six items in which participants indicated how often they take part in activities involving helping others, volunteering, and participating in school organizations. Response options for the first question, which assessed whether youth “do things so that people in the future can have a better life”, ranged from 1 (strongly disagree) to 5 (strongly agree). Response options for the next two questions, which assessed frequency of time youth spent helping others (i.e., friends or neighbors), ranged from 1 (never) to 5 (very often). The last three questions measured frequency of participation in extracurricular activities that served others (e.g., peer advising, school government) and ranged from 1 (never) to 6 (every day). These items are derived from the Profiles of Student Life-Attitudes and Behaviors Survey (PSL-AB; Benson et al., 1998) and the Teen Assessment Project Survey Question Bank (TAP; Small and Rodgers, 1995). Across items, higher scores reflected higher levels of contribution. Cronbach's alpha in the current sample was 0.56 and the mean inter-item correlation was 0.214.

#### Covariates

The potential impact of age and site were controlled by modeling them as covariates. Age was calculated based on participants' self-reported birth month and year. The mean reported age within the analytic subsample was 15.23 (*SD* = 1.94). As noted above, sites were the program sites located in six cities in the United States (Phoenix, AZ; Aurora, CO; Detroit, MI; Cleveland, OH; San Francisco, CA; and St. Louis, MO).

### Procedure

This project was reviewed and approved through the research team's university Institutional Review Board. Informed consent/assent was obtained from all individual participants. Members of the research team traveled to each program site to collect data. To collect cross-sectional data from participants across the six sites, a detailed data collection protocol was used for uniform administration of questionnaires. Directions for completing the questionnaires were shared with participants before they started the survey. Participants were informed that all identifying information would be detached from their survey and kept confidential. Questionnaires were administered via Qualtrics (Qualtrics, Provo, UT) on computers provided by the program, or paper surveys were administered to those without internet access. Participants took ~30 min to complete the questionnaires. After completing the survey, each youth received a $10 gift card. Graduate research assistants entered all paper questionnaire results into a database where they were combined with data collected electronically.

### Analyses

Structural equation modeling (SEM) was used to examine the measurement model depicted by our conceptual model (Figure 1) and to test hypothesized associations between each construct—critical reflection, hopeful future expectations, and mentoring relationship quality—and the outcome of contribution. Because SEM tests relations among latent constructs concurrently while adjusting for measurement error, and it also measures how well complex modes fit the data, it was appropriate for our analysis (Kline, 2016). We completed analyses in Mplus version 8.5 and used the MLR estimator to provide maximum likelihood parameter estimates robust to non-normality (Muthén and Muthén, 2017).

Following Westland (2010) approach for ensuring sufficient sample size in SEM analyses, we determined at 0.80 statistical power and given an a priori alpha level of 0.05, this model and our analytic sample size (*n* = 177) was more than sufficient to detect a standardized effect size of 0.35 or above.

The first step in our analyses was to estimate the measurement model for our hypothesized latent constructs, in order to assess the internal consistency and quality of study measures. Model fit was assessed using the root mean square error of approximation (RMSEA), the comparative fit index (CFI), the Tucker-Lewis index (TLI), and the Standardized Root Mean Square Residual (SRMR). Models with RMSEA values less than or equal to 0.05 are considered to be a very good fit, with values between 0.06 to 0.08 demonstrating adequate fit. Similarly, while CFI and TLI at or above 0.95 are preferred to indicate a well-fitting model, values at or above 0.90 are also generally considered adequate. SRMR values at or below.8 are preferred (Hu and Bentler, 1998, 1999; Kline, 2016). After identifying a well-fitting measurement model, the second step of our analyses, was to test the full structural model to determine the extent to which youths' critical reflection, hopeful future expectations, and mentoring relationship quality related to their contribution, controlling for age and site.

## Results

### Measurement Model

The initial measurement model we specified demonstrated a poor fit, with model fit indices falling outside generally accepted cutoffs for acceptable model fit: RMSEA = 0.063 (90% *CI* = 0.054, 0.072), *CFI* = 0.879, *TL*I = 0.867, and *SRMR* = 0.066. To assess the results of the measurement model for potential sources of poor measurement or model misspecification, we examined item factor loadings for each respective latent construct as well as modification indices (e.g., to determine if shared error covariance among any indicators may have contributed the poor model fit observed; see also Supplementary Table 1 for item-item correlations for all items in this initial measurement model). Through this analysis, we identified two items for removal from the measurement model due to low factor loadings. One item was removed from the mentoring relationship quality construct and one from contribution.

Taking into account semantic and/or conceptual similarity among indicators, along with model modification indices, we also identified four item pairs for which shared error covariance should be estimated (see Table 1). We thus re-specified the measurement model, removing the two items that had low factor loadings and including the shared error covariance estimates for the four identified item pairs. The re-specified measurement model was a good fit to the data: *RMSEA* = 0.054 (90% *CI* = 0.044, 0.064), *CFI* = 0.921, *TLI* = 0.911, and *SRMR* = 0.066. All items loaded significantly and as expected onto their latent construct. Subsequent analyses were based on the adjustments made during this re-specification process.

**Table 1:** Measurement model: Factor loadings for latent variables.

**Latent Variable and Indicators**	**Unstandardized estimate**	***SE***	**Unstandardized estimate / *SE***	**Standardized estimate**
**Critical Reflection**				
How much do you agree with these statements				
(1) Certain racial or ethnic groups have fewer chances to get a good high school education	1.144^*^	0.069	16.484	0.803^*^
(2) Poor children have fewer chances to get a good high school education	1.043^*^	0.076	13.716	0.787^*^
(3) Certain racial or ethnic groups have fewer chances to get good jobs	1.241^*^	0.058	21.512	0.902^*^
(4) Women have fewer chances to get good jobs	1.163^*^	0.070	16.670	0.826^*^
(5) Poor people have fewer chances to get good jobs	1.102^*^	0.068	16.250	0.846^*^
(6) Certain racial or ethnic groups have fewer chances to get ahead	1.283^*^	0.053	24.230	0.920^*^
(7) Women have fewer chances to get ahead	1.211^*^	0.069	17.655	0.857^*^
(8) Poor people have fewer chances to get ahead	1.163^*^	0.063	18.419	0.866^*^
Hopeful Future Expectations				
Think about your futures. What are your chances for the following:				
(1) Graduate from college	0.358^*^	0.060	5.956	0.560^*^
(2) Have a job that pays well	0.470^*^	0.050	90451	0.746^*^
(3) Be healthy	0.524^*^	0.058	9.066	0.725^*^
(4) Be safe	0.657^*^	0.048	13.560	0.899^*^
(5) Be involved in helping other people	0.509^*^	0.062	8.226	0.624^*^
(6) Have friends you can count on	0.470^*^	0.050	90451	0.746^*^
Mentoring Relationship Quality				
Think about the adult mentor you are closest to in this program:				
(1) How close is your relationship with this person	0.500^*^	0.084	5.992	0.659^*^
(2) When I am with this person, I feel bored	0.318^*^	0.078	4.064	0.450^*^
(3) This person says we will do something, but then we don't do it	0.212^**^	0.087	2.441	0.261^**^
(4) I can trust this person	0.290^*^	0.063	4.596	0.405^*^
(5) When something is bugging me, this person listens to me	0.321^*^	0.075	4.297	0.526^*^
(6) This person has good ideas about how to solve problems	0.323^*^	0.066	4.893	0.634^*^
(7) This person talks to me about my future	0.382^*^	0.077	4.981	0.639^*^
(8) This person helps with my schoolwork	0.416 ^*^	0.090	4.637	0.449^*^
Contribution:				
(1) I often do things so that people in the future can have a better life.	0.506^*^	0.065	7.752	0.599^*^
How often do you do the following things:				
(2) Help a friend	0.384^*^	0.081	4.719	0.549^*^
(3) Help a neighbor	0.303^**^	0.119	2.552	0.243^**^
How often do you participate in the following school clubs or activities:				
(4) Volunteering your time (somewhere like at a hospital, daycare center, food bank, youth program, community service agency)	0.455^*^	0.141	3.219	0.367^*^
(5) Mentoring/Peer Advising	0.700^*^	0.156	4.481	0.437^*^
(6) School Government or Other Organization at your School	0.681^*^	0.171	3.969	0.394^*^

*In the respecified measurement model, shared error covariance was estimated for the following items: Hopeful Future Expectations #1 with #2; Critical Reflection #1 with #3; Critical Reflection #2 with #5; and Critical Reflection #4 with #7*.

* *p-value < 0.001*;

** *p-value < 0.01*;

^***^p-value < 0.05; + p-value < 0.10.

### Structural Model

**Figure 2:**
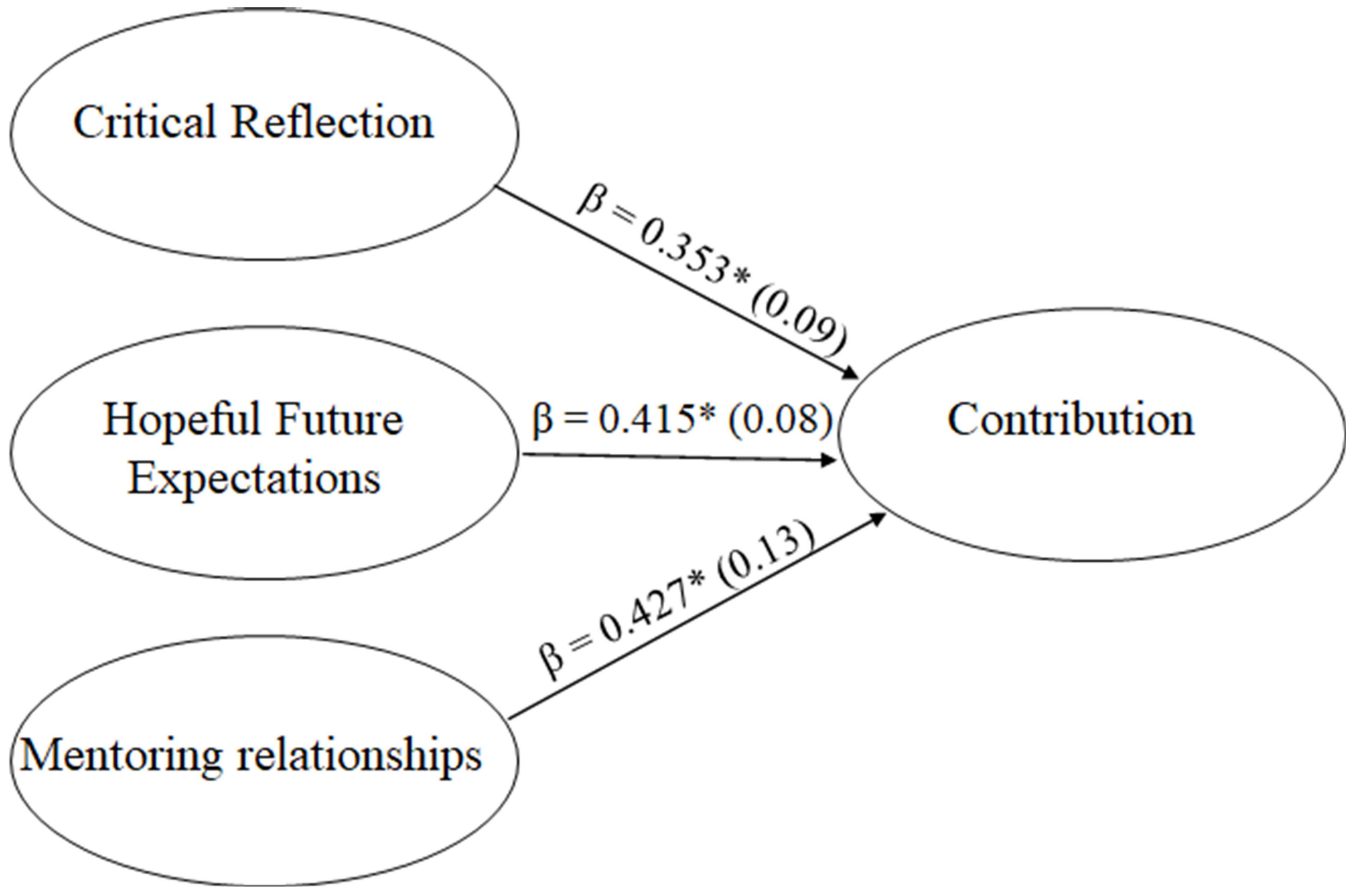
Structural Model. Standardized regression coefficients are depicted for each path, with significant paths at the p < 0.05 level denoted by an asterisk (^*^).

The structural model demonstrated a good fit to the data just as our re-specified measurement model had: *RMSEA* = 0.056 (90% *CI* = 0.046, 0.065), *CF*I = 0.908, *TLI* = 0.897, and *SRMR* = 0.083. In accord with study hypotheses, significant associations were found between critical reflection, hopeful future expectations, and mentoring relationship quality and contribution (βrange = 0.353-0.427). In particular, mentoring relationship quality was most strongly associated with contribution (β = 0.427), followed by youth's hopeful future expectations (β = 0.415), and then critical reflection (β = 0.353). See Figure 2. Further, within the model, mentoring relationship quality, hopeful future expectations, and critical reflection accounted for 56.3% of the variance in youth contribution.

### Exploratory Analysis: Heterogeneity of Effects Across Black and Latinx Participants

Although the sample sizes for the Black subgroup (*n* = 107) and Latinx subgroup (*n* = 70) within our analytic sample of high achieving youth of color were relatively small, we were nonetheless interested in examining if the pattern of associations among study constructs would be consistent across these two subgroups. In specific, the sample size of the Black subgroup was above the minimum sample size of *n* = 94 needed to detect a standardized effect size of 0.35, while the sample size of the Latinx subgroup was just below. For this reason, we conducted what we considered to be exploratory multi-group SEM analyses, with participants grouped based on their ethnic-racial identification of either Black or Latinx. For this multigroup analysis, by default, the means and intercepts of the continuous latent variables in our model were fixed at zero for the Black subgroup and freed for estimation in the Latinx subgroup. The structural path regression coefficients were also freely estimated across groups. Decrements in overall model fit were observed, as expected, based on the smaller sample sizes among the Black and Latinx subgroups: *RMSEA* = 0.081 (90% *CI* = 0.072, 0.090), *CFI* = 0.819*, TLI* = 0.810, *SRMR* = 0.127.

Notwithstanding, there were differences in the associations among study constructs between the two subgroups. Specifically, the relation between critical reflection and contribution was significant for Black youth but not Latinx youth (β*Blac*k = 0.494, β*Latin*x = −0.098). Similarly, the relation between youth's hopeful future expectations was significant for Black but not Latinx youth (β*Black* = 0.356, β*Latinx* = 0.239). Finally, while the path estimate was similar across groups, the relation between mentoring and contribution was also significant only for Black youth, though this was likely due to a larger standard error for the estimate among Latinx youth (β*Black* = 0.449, β*Latin*x = 0.448). See Table 2. While these exploratory results should be interpreted with caution, as noted in our discussion, our preliminary analyses suggest critical reflection and hopeful future expectations may play a substantially different role in the lives of Black versus Latinx youth.

**Table 2:** Heterogeneity of effects by ethnic-racial subgroup.

**Measured Relationship**	**Black**	**Latinx**
Critical Reflection **→**Contribution:	0.49^*^ (0.09)	−0.10 (0.23)
Hopeful Future Expectations **→**Contribution:	0.36^*^ (0.09)	0.24 (0.28)
Mentoring Relationship Quality **→**Contribution:	0.45^*^ (0.12)	0.45 + (0.23)

* *p-value < 0.001*;

^**^p-value < 0.01; ^***^p-value < 0.05; + p-value < 0.10.

## Discussion

Theoretical and empirical work on the development of youth of color from a PYD perspective has been growing in recent years (Travis and Leech, 2014; Clonan-Roy et al., 2016; Bowers et al., 2020); however, there is a need for more PYD work exploring the heterogeneity of experiences within populations of youth of color. It is important to better understand the PYD processes within academically high-achieving youth of color as this work can provide insight into the individual strengths and contextual resources that may have promoted their success in one domain of development but may also be linked to their thriving from a more holistic perspective. As these youth often become leaders within their community (Howard et al., 2016), a better understanding of the factors that are linked to their contribution can enhance empirical and practical work. In the present study, we found that our hypotheses were supported. Controlling for youth age and program site, youth critical reflection, hopeful future expectations, and mentoring relationship quality significantly predicted contribution in a sample of high achieving youth of color who were attending a college preparation afterschool program in six cities across the U.S. Exploratory analyses indicated, however, that these relations were only evidenced in Black youth and not Latinx youth.

Study findings are consistent with prior work framed by PYD models of adolescence derived from relatively homogenous samples of White youth (e.g., Schmid et al., 2011) and samples of high achieving youth of color (e.g., Bowers et al., 2020). The present work, however, provides additional evidence of the links between these assets and contribution, a key marker of thriving, in high achieving youth of color residing in urban communities. Prior research on youth of color residing in urban contexts has indicated that when youth within marginalizing systems critically reflect on social inequities, they are more likely to engage in critical action, civic activities, or political actions (e.g., Diemer and Rapa, 2016). The current work extends these links to more traditional types of contribution such as helping a neighbor, volunteering, and peer mentoring.

The present findings also extend existing research connecting hopeful future expectations to contribution and civic engagement in samples of mostly middle-class White youth (e.g., Callina et al., 2015). Despite the growing interest of researchers and practitioners in the role of hope in promoting PYD, very little empirical work exists that links hope and contribution in adolescents from diverse cultural, ethnic, religious, and socioeconomic backgrounds (Callina et al., 2015). As with critical reflection, much of the extant work in this area focuses on the links between hope and sociopolitical development as compared to more traditional measures of contribution (e.g., Christens et al., 2018). Therefore, these results provide evidence of the potential shared function of hope across ethnic-racial identity groups, socioeconomic status, and context.

In addition to benefits of individual strengths for youth contribution, our results also pointed to mentoring relationship quality as a key contextual resource for supporting contribution. The present findings are consistent with prior work with high achieving youth of color has linked positive-youth adult relationships to academic success (Williams and Bryan, 2013) and more comprehensive measures of thriving (Bowers et al., 2020). The links between mentoring and contribution extend (Bowers et al., 2020) suggestion that mentors promote outcomes such as socially conscious attitudes, sympathy and empathy for others, and stronger connections to others in their mentees. Future research should test whether the full Five Cs model of PYD (Lerner et al., 2015) can be applied to the development of academically high-achieving youth of color.

Although exploratory in nature, our findings indicated that only Black youth exhibited significant relations between the youth assets of critical reflection, hopeful future expectations, and mentoring relationship quality and youth contribution; parallel relations were not evident in the sample of Latinx youth. The current findings suggest that PYD processes may not only be more complex for youth of color and may not follow the same patterns as for White youth (Spencer and Spencer, 2014; Travis and Leech, 2014), but the relation between youth assets and contribution may differ within youth of color as a function of ethnic-racial identification status, among other things. These exploratory findings are consistent with prior work exploring the links between critical reflection and youth outcomes among various ethnic-racial identification groups (Diemer and Rapa, 2016; Godfrey et al., 2019; Tyler et al., 2019). For example, Tyler and colleagues found that critical reflection was negatively related to character, caring, and connection in White youth attending Title 1 schools (U.S. schools that receive supplemental federal funds to serve a student population in which at least 40% of students are considered low income), but not related to any of the Cs in Black youth who attended Title 1 schools. The differential relations that were identified through disaggregation of the data reflect the specificity principle (Bornstein, 2017) and highlight the heterogeneity of experiences of youth—and especially of youth of color, as related to their intersecting social identifies (Crenshaw, 1990; Godfrey and Burson, 2018) and as articulated by RDS models of human development (Lerner et al., 2015). The specificity principle posits that developmental phenomena are to be understood in light of the characteristics of a specific person at a specific time in life in relation to specific characteristics of the context in which they are embedded (Lerner and Chase, 2019).

### Limitations and Future Directions

The primary limitations of the present study are related to the sample size and cross-sectional nature of the data. For example, the relatively small sample size that resulted from disaggregating our data by ethnic-racial identification compelled us to raise a caution when interpreting the results related to our exploratory subgroup analyses. In addition, a larger sample size would allow for a greater consideration of the processes that are related to ethnic-racial identification as compared to using ethnic-racial identification as a grouping variable (Williams and Deutsch, 2016). A larger sample would also provide for consideration of additional dimensions of marginalization such as gender, immigrant status, and language status (Lerner et al., 2013; Causadias and Umaña-Taylor, 2018)—all important facets of social identity that shape the way individuals experience marginalizing systems.

As the study was cross-sectional, we were not able to determine directionality of the relations between the constructs of interest. We hypothesized that youth strengths and resources predicted levels of youth contribution; however, it is also expected that through participating in acts of contribution, youth may be engaged to participate in experiences that promote critical reflection, hopeful expectations, and connections with adults. Indeed, PYD models posit that processes are non-recursive so youth contribution feeds back to benefit the individual and his or her context (Travis and Leech, 2014; Lerner et al., 2015). Therefore, future work should collect longitudinal data from larger samples of diverse youth.

Care must also be taken with applying these findings beyond the population of high achieving youth of color residing in urban areas who participate in college preparation programming. Therefore, we suggest caution when considering the validity of these findings for youth of color in general or for high-achieving youth of color who may not been engaged in this type of programming. This specific college preparation program, BHGH, also emphasizes participating in service opportunities as a core part of their programming. Therefore, the level and types of contribution with which youth were engaged, and the processes that promote that engagement, may not apply to other populations of youth of color. Additional research within more representative populations of high achieving youth of color recruited from schools and a diversity of afterschool programs is recommended.

The study is also limited in its operationalization of contribution based on a measure derived from the 4-H Study of PYD (e.g., Bowers et al., 2014). This measure was developed based on a sample of mostly White, middle class youth (Spencer and Spencer, 2014; Hershberg et al., 2015) and included items that index more traditional forms of contribution such as helping others, mentoring, and volunteering. These types of contribution may not be as readily accessible or valued by youth of color residing in urban areas (Ginwright and Cammarota, 2002; Hope and Jagers, 2014; Hershberg et al., 2015). The comparatively low reliability statistic may be a reflection of this concern with the population of interest in this study. Therefore, future work should include both traditional and other types of contribution such as organizing, protesting, and youth activism (i.e., activities more reflective of critical action). Including other measures of contribution will help us continue developing our understanding of contribution and associated factors among high-achieving youth of color.

Finally, future work should include qualitative methods to complement the quantitative method utilized here, as a mixed-method approach will enhance our understanding of the processes that underly the links between these youth assets and contribution such as how youth value contribution in their lives and what antecedents influence youth to take action (e.g., Hershberg et al., 2015; Tyler et al., 2020). A qualitative approach would also allow for an exploration of the processes of mentoring through a more critical lens as there are concerns that mentoring programs frequently reproduce existing systemic inequalities and reaffirm the status quo (Albright et al., 2017; Sánchez et al., 2021).

### Implications

The present study identified several factors at the individual (critical reflection and hopeful future expectations) and contextual (mentoring relationship quality) level linked to contribution in high-achieving youth of color residing in urban contexts. Each of the identified assets are sensitive to programming and or training (e.g., Ginwright and James, 2002; Herrera et al., 2013; Callina et al., 2015). Therefore, the results point to several malleable strengths that practitioners and programs can build and leverage to enhance the likelihood youth will contribute to their communities. Programs that aim to increase active civic engagement in youth might first implement hope enhancement strategies (Callina et al., 2015) which promote goal-directed skills, positive future orientations, and connectedness to peers and adults. Those who work with youth can be encouraged to incorporate SJYD principles into their work, such as providing space for critical reflection, empowering youth, and celebrating youth culture (Ginwright and James, 2002).

The differential relations between the assets and contribution for Black versus Latinx youth identified in the present study suggest that a better understanding of both the specific and the common relations between these youth assets and youth contribution will help scholars and practitioners identify culturally relevant ways to promote thriving in diverse youth. Therefore, programs and practices aimed at promoting contribution in youth of color by strengthening their individual or contextual assets such as critical reflection, hopeful future expectations, or mentoring relationship quality should be tailored to meet the individual needs of young people that build off their strengths. For example, training adults who work with youth in cultural empathy or to support the ethnic/racial identity of youth of color could influence mentoring relationship quality for diverse youth (Sánchez et al., 2019). Research suggests that when mentors are trained in SJYD principles, they not only become more aware of the needs of their mentees, but they also grow in their own critical consciousness (Anderson et al., 2018).

Finally, extant work also suggests that youth across the developmental continuum may meaningfully engage in critical reflection and critical action (e.g., Rapa et al., 2020b). Therefore, finding links between critical reflection and types of contribution that are more frequently offered in schools and youth programs may provide new insights for how to motivate youth to engage in service opportunities and activities that may, in turn, lead to enhanced critical reflection and action.

### Conclusion

Despite its limitations, the present study still contributes to the limited body of research on youth of color residing in urban environments from a PYD perspective. While such youth are often leaders of their community and they succeed under the oppressive forces of systemic inequities, the strengths and successes of academically high achieving youth of color are frequently overlooked (Olszewski-Kubilius and Clarenbach, 2012). The present study has identified individual strengths and contextual resources that are malleable and can be leveraged to promote positive outcomes in high achieving youth of color that go beyond academic success. In addition, the exploratory findings point to the importance of considering the heterogeneity of experiences within youth of color when identifying ways to optimize youth contributions to their families, schools, and communities. Nurturing the strengths of critical reflection and hopeful future expectations and connecting youth to caring and concerned mentors will support youth developing to their full potential and becoming agents of social change.

## Data Availability Statement

The datasets generated for this study are available on request to the corresponding author.

## Ethics Statement

The studies involving human participants were reviewed and approved by Clemson University. Written informed consent to participate in this study was provided by the participants' legal guardian/next of kin.

## Author Contributions

EB is the principal investigator of the study and oversaw conceptualization of the study, funding acquisition, and writing of the original draft. CB conducted data analysis and wrote results, generated tables and figures, contributed to the literature review and editing of the manuscript. LR supported data analysis, contributed to the literature review, and reviewed and edited the manuscript. AS reviewed and edited manuscript, completed reference check, and supported table and figure development. All authors contributed to the article and approved the submitted version.

## Conflict of Interest

The authors declare that the research was conducted in the absence of any commercial or financial relationships that could be construed as a potential conflict of interest.
