# Supplementary material for: Predicting Contribution in High Achieving Black and Latinx Youth: The Role of Critical Reflection, Hope, and Mentoring
Source: Front Psychol. 2021 Jul 7;12:681574. doi: 10.3389/fpsyg.2021.681574 (PMC8292631; doi:10.3389/fpsyg.2021.681574)
Supplement: Supplementary file 1 [file Data_Sheet_1.PDF]

Table S.1

Item-item Correlations

	Ment 1	Ment 2	Ment 3	Ment 4	Ment 5
Ment 1	1.00				
Ment 2	.463***	1.00			
Ment 3	0.068	.276***	1.00		
Ment 4	.319***	.169*	.167*	1.00	
Ment 5	.283***	.258**	0.131	.342***	1.00
Ment 6	.329***	.171*	.294***	.197*	.459***
Ment 7	.443***	.195*	0.061	.185*	.182*
Ment 8	.258**	0.083	0.083	0.068	.223**
Cont 1	.271***	.157*	0.078	.199*	0.123
Cont 2	.186*	.259**	-0.020	.199*	.165*
Cont 3	0.038	-0.008	-0.004	0.010	0.143
Cont 4	0.135	0.129	-0.102	-0.023	0.057
Cont 5	0.151	0.023	0.018	0.095	0.055
Cont 6	0.136	0.064	0.097	0.032	0.039
HFE 1	.154*	0.038	0.010	.244**	.197*
HFE 2	0.052	0.019	0.004	0.145	.188*
HFE 3	0.069	0.038	-0.027	.161*	0.088
HFE 4	0.054	0.049	0.067	0.076	0.116
HFE 5	0.132	0.098	-0.031	0.022	0.117
HFE 6	.181*	.264**	0.113	0.033	.207**
CR 1	-0.001	-0.045	-.165*	0.021	-0.115
CR 2	-0.041	-0.092	-0.073	-0.049	-0.041
CR 3	-0.045	-0.073	-0.108	0.021	-0.088
CR 4	-0.048	-0.124	-0.125	0.091	-0.075
CR 5	-0.091	-0.110	-0.068	-0.019	-0.153
CR 6	-0.002	0.004	-0.038	0.083	-0.029
CR 7	-0.036	0.021	-0.067	0.095	-0.070
CR 8	-0.025	0.020	-0.049	-0.012	-0.097

***p < .001; **p < .01; *p < .05

Note. Ment = mentoring; Cont = contribution; HFE = hopeful future expectations; CR = critical reflection.

Table S.1 Continued

	Ment 6	Ment 7	Ment 8	Cont 1	Cont 2
Ment 6	1.00				
Ment 7	.488***	1.00			
Ment 8	.273***	.438**	1.00		
Cont 1	0.049	.174*	0.125	1.00	
Cont 2	0.126	.171*	0.021	.360***	1.00
Cont 3	0.106	0.010	0.018	0.052	.222**
Cont 4	0.041	0.073	0.102	.184*	0.105
Cont 5	0.001	0.086	0.026	.233**	.203**
Cont 6	0.006	0.074	-0.004	.153*	0.112
HFE 1	0.052	0.020	0.015	.267***	.168*
HFE 2	0.079	-0.082	-0.061	.256**	.254**
HFE 3	0.077	-0.095	-0.032	.165*	.209**
HFE 4	0.124	-0.026	0.050	.213**	.152*
HFE 5	.193*	0.038	0.019	.216**	.208**
HFE 6	0.116	-0.034	0.039	.323***	.268***
CR 1	-.163*	-0.063	-.204*	0.130	0.122
CR 2	-0.044	-0.083	-.203*	0.073	0.093
CR 3	-0.150	-0.099	-.191*	.168*	.192*
CR 4	-.159*	-0.050	-.195*	0.066	0.143
CR 5	-0.122	-0.098	-.268**	0.107	0.139
CR 6	-0.085	-0.005	-.181*	.162*	.281***
CR 7	-0.152	-0.050	-.167*	.163*	.256**
CR 8	-.159*	-0.053	-.203*	.180*	.168*

***$p < .001$; **$p < .01$; *$p < .05$

Note. Ment = mentoring; Cont = contribution; HFE = hopeful future expectations; CR = critical reflection.

Table S.1 Continued

	Cont 3	Cont 4	Cont 5	Cont 6	HFE 1
Cont 3	1.00				
Cont 4	0.083	1.00			
Cont 5	0.075	.295***	1.00		
Cont 6	0.117	.291***	.297***	1.00	
HFE 1	0.146	0.027	0.078	0.102	1.00
HFE 2	0.007	0.116	0.132	0.146	.649***
HFE 3	0.059	0.021	0.136	0.126	.528***
HFE 4	0.017	0.079	.200**	.169*	.457***
HFE 5	0.084	0.106	.186*	0.147	.339***
HFE 6	0.068	0.105	.172*	.150*	.265***
CR 1	0.036	0.128	0.008	.154*	-0.015
CR 2	.173*	0.094	0.016	.162*	-0.040
CR 3	0.076	.158*	0.078	.172*	0.020
CR 4	0.108	0.069	0.027	0.060	0.026
CR 5	0.077	0.024	-0.051	0.107	0.049
CR 6	0.117	.190*	0.101	.165*	0.078
CR 7	0.131	0.113	0.069	.187*	0.076
CR 8	0.076	0.122	0.112	.216**	0.040

****p* < .001; ***p* < .01; **p* < .05

Note. Ment = mentoring; Cont = contribution; HFE = hopeful future expectations; CR = critical reflection.

Table S.1 Continued

	HFE 2	HFE 3	HFE 4	HFE 5	HFE 6
HFE 2	1.00				
HFE 3	.594***	1.00			
HFE 4	.655***	.650***	1.00		
HFE 5	.413***	.393***	.587***	1.00	
HFE 6	.380***	.283***	.486***	.351***	1.00
CR 1	0.068	0.093	0.017	0.027	0.011
CR 2	-0.009	0.071	-0.067	0.073	0.022
CR 3	0.053	0.108	-0.017	0.036	-0.037
CR 4	0.044	0.100	-0.034	0.048	-0.070
CR 5	0.049	0.089	-0.048	0.011	-0.008
CR 6	0.099	0.084	-0.016	0.046	0.002
CR 7	0.099	0.131	0.007	0.025	0.061
CR 8	0.063	0.116	-0.034	0.026	0.042

***$p < .001$; **$p < .01$; *$p < .05$

Note. Ment = mentoring; Cont = contribution; HFE = hopeful future expectations; CR = critical reflection.

Table S.1 Continued

	CR 1	CR 2	CR 3	CR 4	CR 5
CR 1	1.00				
CR 2	.696***	1.00			
CR 3	.830***	.729***	1.00		
CR 4	.692***	.701***	.740***	1.00	
CR 5	.643***	.764***	.766***	.760***	1.00
CR 6	.755***	.680***	.857***	.751***	.748***
CR 7	.700***	.600***	.746***	.804***	.721***
CR 8	.663***	.754***	.755***	.678***	.778***

***$p < .001$; **$p < .01$; *$p < .05$

Note. Ment = mentoring; Cont = contribution; HFE = hopeful future expectations; CR = critical reflection.

Table S.1 Continued

	CR 6	CR 7	CR 8
CR 6	1.00		
CR 7	.811***	1.00	
CR 8	.792***	.759***	1.00

***p < .001; **p < .01; *p < .05

Note. Ment = mentoring; Cont = contribution; HFE = hopeful future expectations; CR = critical reflection.
